# Impact of plant sex on endophytic microbial diversity and community assembly in dioecious species

**DOI:** 10.1128/spectrum.00976-26

**Published:** 2026-09-22

**Authors:** Meiqin Mao, Yutong Wu, Linxin Ye, Xinyi Su, Jiaye Zhang, Shufen Cui, Chenmiao Wang, Shaokai Xu, Yuxin Jian, Luping Qin, Bo Zhu

**Affiliations:** 1 School of Pharmaceutical Sciences, Zhejiang Chinese Medical University 70571, Hangzhou, China; 2 School of Business, Lishui University 117807, Lishui, China; 3 Zhejiang Key Laboratory of Chinese Medicine Modernization, Zhejiang Chinese Medical University 70571, Hangzhou, China; Instituto de Ecología, A.C. (INECOL), Michoacán, Mexico

**Keywords:** dioecious plants, plant endophytes, bacterial and fungal communities

## Abstract

Dioecious plants show marked sexual dimorphism in morphology, physiology, defense, and life-history allocation, yet differences in endophytic microbiomes remain understudied. We compared endophytic bacterial and fungal communities between male and female individuals of four dioecious species—*Eucommia ulmoides*, *Taxus wallichiana* var. *mairei*, *Ginkgo biloba*, and *Morus alba*—by high-throughput sequencing of bark (*E. ulmoides* and *T. wallichiana* var. *mairei*) and leaves (*G. biloba* and *M. alba*). Sex significantly influenced the assembly of bacterial and fungal communities in all species. Bacterial alpha diversity (Shannon index) was significantly higher in males than females for *E. ulmoides*, *T. wallichiana* var. *mairei*, and *M. alba*. The bacterial phylum Spirochaetota was only detected in male samples of *E. ulmoides*, *G. biloba*, and *M. alba*, while the fungal genera *Albophoma* and *Striaticonidium* were detected only in female samples across all four species. Acidobacteria were significantly enriched in males of *E. ulmoides* and *T. wallichiana* var. *mairei*, whereas the fungal genus *Sporidesmium* was more abundant in females of *T. wallichiana* var. *mairei* and *M. alba*. Despite these sex-driven differences, dominant endophytic composition was similar between sexes: Actinobacteriota and Proteobacteria were the most prevalent bacterial phyla, whereas Ascomycota and Basidiomycota were the most prevalent fungal phyla across all samples. Co-occurrence analysis indicated stronger links between Actinobacteriota or Proteobacteria and other phyla than among remaining phyla. These results enhance understanding of sex-associated endophytic communities in dioecious plants and may inform studies on plant sex differentiation and potential plant–microbiome associations.

**IMPORTANCE:**

Plant sex influences physiology, defense, and resource allocation, but its effects on internal microbial communities remain poorly understood. We compared endophytic bacteria and fungi in male and female Eucommia ulmoides, Taxus wallichiana var. mairei, Ginkgo biloba, and Morus alba using high-throughput sequencing and found consistent sex-associated differences in microbial diversity and specific taxa (e.g., Spirochaetota enriched in males; Albophoma and Striaticonidium enriched in females). These results indicate that sex is a reproducible driver of endophyte community assembly across diverse hosts. Recognizing sex-specific microbiomes has practical implications for conservation, forest and crop health management, and targeted bioprospecting, and underscores the need to include host sex as a key factor in ecological, functional, and evolutionary studies of plant–microbe interactions.

## INTRODUCTION

Dioecious plants have developed from hermaphroditic ancestors and are widely distributed across plant lineages, ranging from bryophytes and gymnosperms to angiosperms (1–3). Dioecious angiosperms, characterized by unisexual flowers borne on separate male and female individuals, comprise 987 genera and 175 families, with approximately 15,600 species—accounting for 5%–6% of all angiosperm species (1–3). These plants play a crucial role in conserving species diversity and maintaining ecosystem stability. Male and female plants exhibit pronounced sexual dimorphism in morphology, physiology, defense mechanisms, and life-history allocation patterns (4, 5). Compared with female plants, males generally display greater tolerance to environmental stresses such as drought, salinity, heavy metals, and nutrient deficiency. For instance, under nutrient-limited conditions, male *Populus cathayana* plants tend to allocate more resources to aboveground growth, whereas females prioritize the biosynthesis of flavonoids and other secondary metabolites (6, 7). This sex-specific resource allocation directly results in significant differences in metabolic profiles between the sexes. Female *Tinospora cordifolia* exhibited higher contents of total phenols, total tannins, and starch than male plants (8). Similarly, female *Juniperus communis* allocated more resources to storage and defense, leading to significantly higher concentrations of carbohydrates, carbon, and phenolic compounds in their needles compared with males (9). In *Cannabis sativa*, female flowers are the primary sites for the synthesis and accumulation of cannabinoids such as tetrahydrocannabinol and cannabidiol, with cannabinoid levels markedly higher in females than in males (10, 11). Moreover, volatile emissions from willow catkins differ between sexes: male flowers emit greater total terpenoid quantities, whereas female flowers produce more benzenoids (12).

Endophytes are microorganisms that colonize plant tissues without causing apparent disease symptoms at any stage of the host’s life cycle, primarily comprising fungi and bacteria. They fulfill important ecological roles in enhancing plant resilience to various abiotic stresses; for example, certain plant growth-promoting bacteria isolated from internal tissues can improve plant tolerance to high salinity, extreme temperatures, and drought (13, 14). Furthermore, endophytes substantially influence the accumulation of plant secondary metabolites, and some are capable of directly synthesizing bioactive compounds that are identical or structurally related to those of their hosts. For instance, the endophytic fungus *Taxomyces andreanae* produces paclitaxel—the same anticancer compound found in its host, the Pacific yew (15). Sex-specific variations in plant metabolites may act as a selective filter shaping endophytic community assembly. Under different nitrogen regimes, root tannin concentrations were consistently higher in male than in female *P. cathayana*. Correlation analyses further revealed that root tannin content significantly influenced the relative abundances of Chloroflexi, Actinobacteriota, and Proteobacteria, as well as bacterial Shannon diversity (16). Sex-driven physiological and metabolic differences can shape distinct endophytic microbiomes. In plant–microbe interactions, secondary metabolites such as phenolics and flavonoids often act as selective filters, enriching tolerant microbial taxa while inhibiting susceptible ones (17). For example, defensive compounds like tannins possess strong broad-spectrum antimicrobial properties (18). Therefore, differences in tannin accumulation between male and female tissues are likely to create distinct biochemical microenvironments. Tissues with higher tannin concentrations may restrict the colonization of generalist endophytes, potentially leaving open niches for specific taxa capable of tolerating or metabolizing these compounds. In addition, sex-dependent differences in floral volatile emissions, such as the differential release of antimicrobial terpenes and benzenoids, may directly modulate floral microbiomes (19). Therefore, we propose that pronounced metabolic dimorphism fundamentally generates distinct selective pressures in male and female tissues, thereby inevitably shaping the recruitment and assembly of their endophytic microbiomes.

*Eucommia ulmoides*, *Taxus wallichiana* var. *mairei*, *Ginkgo biloba*, and *Morus alba* are dioecious species of considerable medicinal, ecological, and economic value. *E. ulmoides*, a model system for studying sex differentiation, produces eucommia rubber throughout nearly its entire body and yields durable, decay-resistant timber, contributing to its high economic worth; it has also been used as a renowned medicinal plant in China for over 2,000 years (20). *T. wallichiana* var. *mairei*, a precious and endangered species with a history spanning approximately 2.5 million years—often termed “plant gold”—plays a crucial role in biodiversity conservation (21). It synthesizes a range of natural compounds with anticancer properties, most notably paclitaxel. *G. biloba*, considered a “living fossil,” exhibits high resistance to atmospheric pollutants such as sulfur dioxide, making it an ideal urban street tree (22). In addition, its leaf extracts are used in adjunctive treatment of Alzheimer’s disease and atherosclerosis (23, 24). *M. alba* is a vital economic species for sericulture, with edible fruits and medicinally valuable bark and leaves (25). Notably, sexual dimorphism significantly influences their metabolic profiles. In *E. ulmoides*, males show significant differences in total phenolic acid and volatile compound content compared with females (25, 26). Alpha-linolenic acid and its metabolites in the bark, along with flavonoids in the leaves, represent the primary differentially accumulated metabolites between sexes (27). *G. biloba* also exhibits sexually dimorphic metabolite production; for example, chalcone levels are significantly higher in young female leaves, whereas male leaves typically possess greater phenolic content and antioxidant activities (28). In *M. alba*, male inflorescences contain more unique genes, with numerous highly expressed genes enriched in pathways involved in synthesizing defensive secondary metabolites such as phenylpropanoids, cutin, and suberin (29). Despite these advances in understanding physiological and metabolic dimorphism, knowledge of differences in endophytic microbial communities between male and female individuals of these species remains limited.

In this study, two gymnosperm species—*T*. *wallichiana* var. *mairei* and *G. biloba*—and two angiosperm species—*E*. *ulmoides* and *M. alba*—were examined to compare endophytic bacterial and fungal composition between sexes using high-throughput sequencing. Sex is an important factor influencing endophytic microbiomes, but plant tissue type also strongly shapes microbial community composition and can affect cross-species comparisons when different tissues are sampled. This study therefore aims to identify sex-associated microbial taxa and assess whether any such taxa are shared among the dioecious species examined. The findings improve understanding of sex-related endophytic variation in dioecious plants and may provide a basis for further investigation of plant–microbiome interactions in an evolutionary context.

## MATERIALS AND METHODS

### Plant sampling

Plant materials from four dioecious species were collected from distinct geographic locations in China: *E. ulmoides* bark from Zunyi City, Guizhou Province (27°43′31.87″N, 106°52′50.69″E); *T. wallichiana* var. *mairei* bark from Lishui City, Zhejiang Province (28°26′58.5″N, 119°54′28.7″E); *G. biloba* leaves from Pizhou City, Jiangsu Province (34°37′48″N, 119°59′24″E); and *M. alba* leaves from Xiushui City, Jiangxi Province (29°01′48″N, 114°33′00″E). All samples were obtained from both male and female individuals. For bark samples (*E. ulmoides* and *T. wallichiana* var. *mairei*), stem segments were excised from the main trunk at a standardized height (~1.2–1.5 m above ground) with a sterile pruning shear; bark was peeled with a sterile scalpel and cut into 1 cm^2^ pieces. For leaf samples (*G. biloba* and *M. alba*), fully expanded, healthy leaves were collected from the middle canopy using sterile scissors. All tools were sterilized with 75% ethanol between trees to prevent cross-contamination. Only trees with similar diameter at breast height and no visible signs of disease or pest damage were sampled. Thirty biological replicates were collected for each species–sex combination, yielding 240 samples in total. Species identity and sex were confirmed by Professor Qiaoyan Zhang (Zhejiang Chinese Medical University). Samples were kept chilled in an ice box during transport to the laboratory, rinsed with sterile ddH_2_O, and surface-sterilized sequentially with 75% ethanol for 1 min, 2% fresh bleach for 3 min, and 75% ethanol for 1 min, followed by a 30-s rinse in sterile ddH_2_O. Samples were stored at −80°C. Sterilization efficacy was checked by plating the final rinse water on nutrient agar; no microbial growth was observed after 48 h.

### DNA extraction, PCR amplification, and high-throughput DNA sequencing

Microbial genomic DNA was extracted from plant tissues using the FastDNA Spin Kit (Omega Bio-tek, Norcross, GA, USA) according to the manufacturer’s instructions (30). DNA integrity was verified on a 1% agarose gel, and DNA concentration and purity were determined using a NanoDrop 2000 UV-vis spectrophotometer (Thermo Fisher Scientific, Wilmington, DE, USA). The 16S ribosomal RNA (rRNA) gene (V3–V4 region) was amplified using the primer pair 799F (5′-AACMGGATTAGATACCCKG-3′) and 1193R (5′-ACGTCATCCCCACCTTCC-3′), while the ITS region was amplified using ITS1F (5′-CTTGGTCATTTAGAGGAAGTAA-3′) and ITS2R (5′-GCTGCGTTCTTCATCGATGC-3′) with an ABI GeneAmp 9700 PCR thermocycler (Applied Biosystems, Carlsbad, CA, USA) (31). The 16S rRNA gene PCR amplification protocol consisted of an initial denaturation at 95°C for 3 min, followed by 13 cycles of denaturation at 95°C for 30 s, annealing at 55°C for 30 s, and extension at 72°C for 45 s, with a final extension at 72°C for 10 min and a hold at 10°C. ITS region amplification followed the same thermal profile but with 35 cycles. The PCR mixture contained 5× TransStart FastPfu buffer (4 μL), 2.5 mM dNTPs (2 μL), forward primer (5 μM, 0.8 μL), reverse primer (5 μM, 0.8 μL), TransStart FastPfu DNA polymerase (0.4 μL), 10 ng template DNA, and nuclease-free ddH_2_O to a final volume of 20 μL (32). PCR products were excised from a 2% agarose gel and purified using the AxyPrep DNA Gel Extraction Kit (Axygen Biosciences, Union City, CA, USA) per the manufacturer’s instructions, then quantified using a Quantus Fluorometer (Promega, Madison, WI, USA). Purified amplicons were pooled in equimolar concentrations and subjected to paired-end sequencing on an Illumina NextSeq 2000 platform (Illumina, San Diego, CA, USA) following standard protocols provided by Majorbio Bio-Pharm Technology Co. Ltd. (Shanghai, China) (33).

### Sequence processing and filtering

Raw FASTQ files were demultiplexed using an in-house Perl script and subsequently quality-filtered with fastp (v0.19.6) and merged using FLASH (v1.2.7) under the following criteria: (i) reads were truncated at any site where the average quality score over a 50-bp sliding window fell below 20; truncated reads shorter than 50 bp and those containing ambiguous nucleotides were discarded; (ii) only overlapping sequences longer than 10 bp were assembled based on their overlap (34), with a maximum mismatch ratio of 0.2 in the overlapping region; unassembled reads were discarded; (iii) samples were distinguished by barcode and primer sequences, with sequence orientation adjusted accordingly—exact barcode matching and up to two nucleotide mismatches in primer matching were permitted. The resulting high-quality sequences were clustered into operational taxonomic units (OTUs) at 97% sequence similarity using UPARSE (v7.1) (35). Bacterial OTUs were annotated with the RDP classifier against the SILVA 16S rRNA database (v138.1) using a 70% confidence threshold, and community composition was profiled at multiple taxonomic levels. Fungal OTUs were annotated identically using the UNITE database (v9.0). The most abundant sequence in each OTU was used as the representative sequence. Chloroplast and mitochondrial sequences were removed from the OTU table. To reduce the effect of unequal sequencing depth on alpha- and beta-diversity analyses, 16S rRNA and ITS counts were rarefied to the size of the smallest sample (36).

### Data analysis

Rarefaction curves were generated using R (v3.3.1). The Shannon index, a measure of alpha diversity, was calculated for both bacterial and fungal communities and visualized using Mothur (v1.30.2) (37). Differences in Shannon indices among treatments were assessed using Welch’s *t*-test with *post hoc* comparisons. Community composition and relative abundances of bacterial and fungal taxa between males and females were compared across *E. ulmoides* and *T. wallichiana* var. *mairei* barks and *G. biloba* and *M. alba* leaves using R (38). Taxonomic bar plots were generated based on the mean relative abundances calculated across replicate samples. Venn diagrams were used to illustrate common and unique bacterial phyla and fungal genera among sexes and host species. To evaluate the effect of sex on beta diversity, nonmetric multidimensional scaling (NMDS) based on Bray–Curtis distances was performed using R and the vegan package (v2.4.3) (39). Differential abundance analysis between sexes was performed using ANCOM-based approaches. Volcano plots were generated in R to visualize sex-associated differences in microbial taxa in the barks of *E. ulmoides* and *T. wallichiana* var. *mairei*, as well as in the leaves of *G. biloba* and *M. alba*. Co-occurrence networks of microbial communities for males and females across *E. ulmoides* and *T. wallichiana* var. *mairei* barks and *G. biloba* and *M. alba* leaves were constructed. Non-random co-occurrence patterns were detected for both bacterial and fungal communities. Analysis was limited to the top 50 most abundant taxa at the class level. Associations were calculated using Spearman’s rank correlation in R, and only correlations with |*r*| ≥ 0.5 and an adjusted *P* < 0.05 (Benjamini–Hochberg FDR) were retained. Networks were visualized in Gephi (40). Functional predictions (KEGG and COG pathways, plus enzyme functions) were generated with PICRUSt2 (http://huttenhower.sph.harvard.edu/galaxy), which infers gene content from OTU phylogeny and reference databases (41).

## RESULTS

### Analysis of sequencing results and alpha diversity

A total of 4,694,884 valid 16S rRNA sequences and 15,394,880 valid ITS sequences were obtained from 240 samples. After quality filtering, the 16S rRNA data set yielded a mean of 19,562 sequences per sample (minimum: 10,046) with an average base count of 7,368,920, whereas the ITS data set yielded 64,145 sequences per sample (minimum: 32,474) with an average base count of 15,029,584. Clustering at 97% identity generated 3,452 bacterial OTUs and 5,741 fungal OTUs (Tables S1 and S2). Rarefaction curves for both markers approached saturation, indicating that sampling captured nearly the full extent of bacterial and fungal community diversity. All rarefaction curves plateaued, confirming that the sequencing depth was sufficient to represent the majority of species present in samples (Fig. S1).

**Fig 1:**
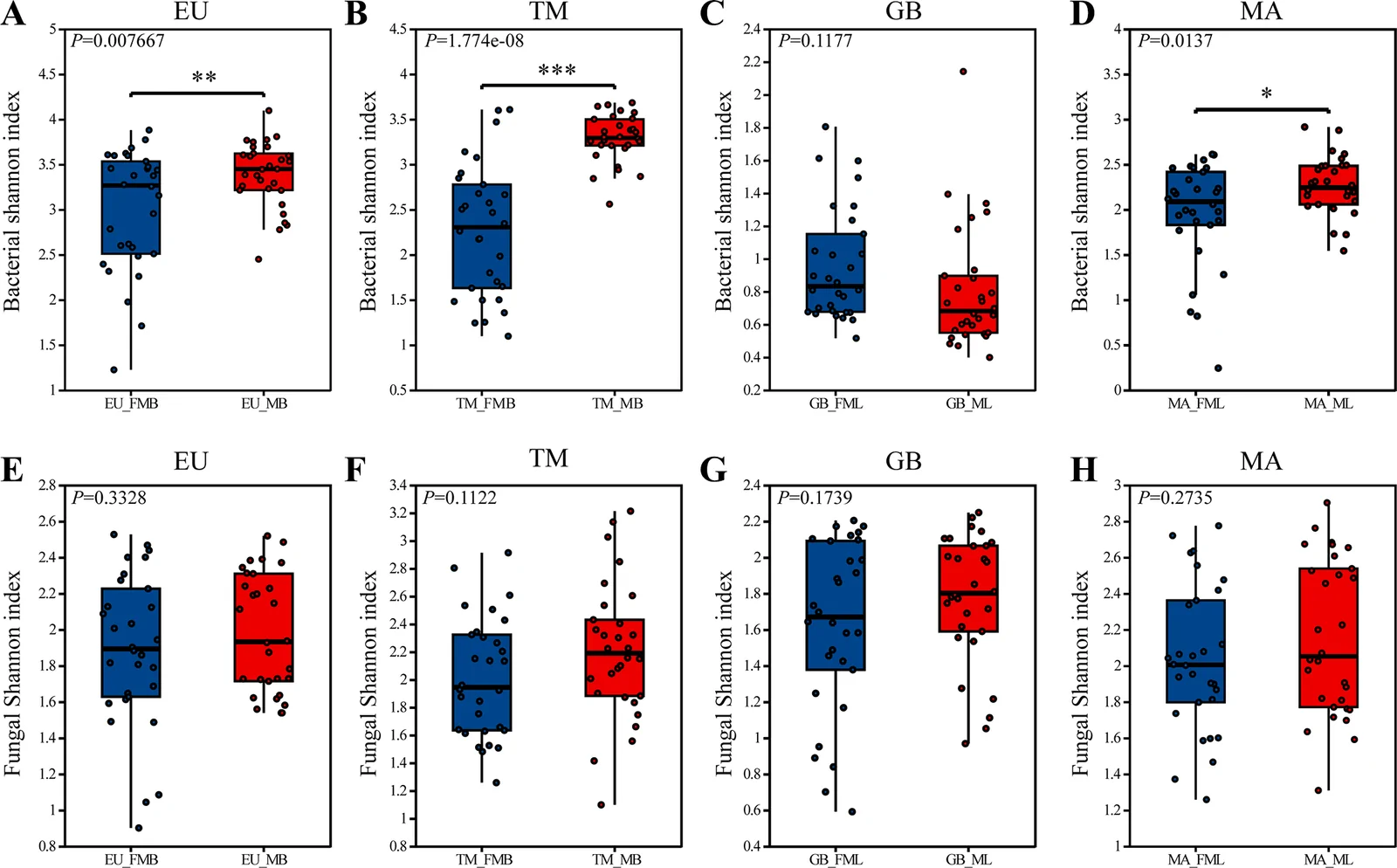
Analysis of alpha diversity index for bacteria (**A–D**) and fungi (**E–H**) across samples at the genus level. Significant differences in alpha diversity between sexes are indicated by asterisks (Welch’s *t*-test; *P < 0.05; **P < 0.01; and ***P < 0.001). EU_FMB, female *E. ulmoides* bark; EU_MB, male *E. ulmoides* bark; TM_FMB, female *T. wallichiana* var. *mairei* bark; TM_MB, male *T. wallichiana* var. *mairei* bark; GB_FML, female *G. biloba* leaves; GB_ML, male *G. biloba* leaves; MA_FML, female *M. alba* leaves; and MA_ML, male *M. alba* leaves.

The Shannon index was used to assess the alpha diversity of endophytic bacterial and fungal communities. In this study, the alpha diversity of bacterial communities in *E. ulmoides* and *T. wallichiana* var. *mairei* barks and *M. alba* leaves was significantly influenced by plant sex, with males exhibiting higher Shannon index values than females. Specifically, the bacterial Shannon index in *T. wallichiana* var. *mairei* male bark (3.2859 ± 0.26258) was significantly higher than that in female bark (2.2656 ± 0.73195) (Fig. 1A through D). In contrast, no significant differences were observed in alpha diversity (Shannon index) of fungal communities between males and females in *E. ulmoides* and *T. wallichiana* var. *mairei* barks or *G. biloba* and *M. alba* leaves (Fig. 1E through H).

### Analysis of endophytic bacterial and fungal community composition between males and females across medicinal plants

**Fig 2:**
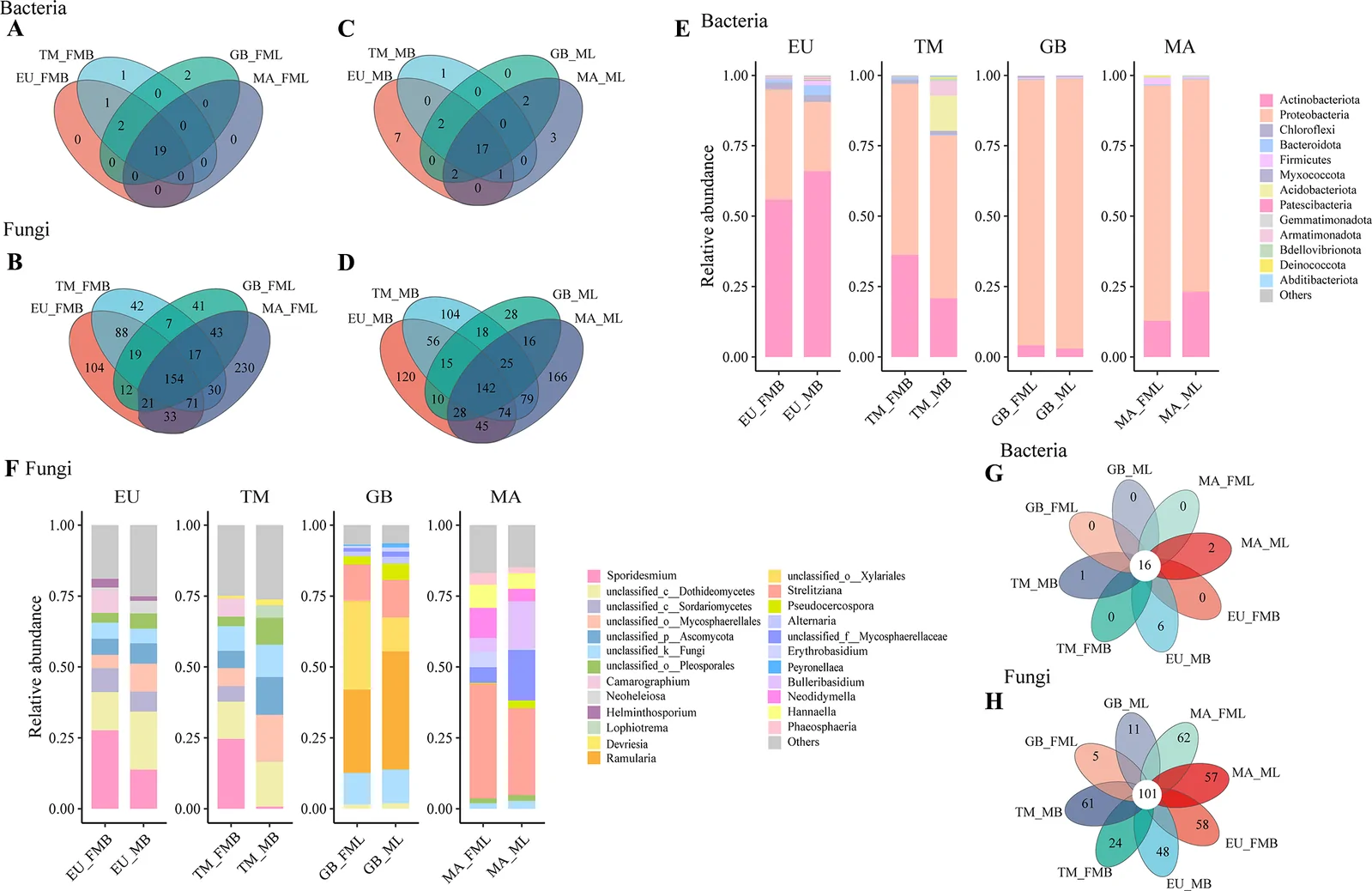
Venn diagrams showing the shared and unique bacterial phyla (**A, C, and G**) and fungal genera (**B, D, and H**) among male and female samples of *E. ulmoides*, *T. wallichiana* var. *mairei*, *G. biloba*, and *M. alba*. Composition and relative abundances of (**E**) bacterial phyla and (**F**) fungal genera in male and female samples across *E. ulmoides*, *T. wallichiana* var. *mairei*, *G. biloba*, and *M. alba*. EU_FMB, female *E. ulmoides* bark; EU_MB, male *E. ulmoides* bark; TM_FMB, female *T. wallichiana* var. *mairei* bark; TM_MB, male *T. wallichiana* var. *mairei* bark; GB_FML, female *G. biloba* leaves; GB_ML, male *G. biloba* leaves; MA_FML, female *M. alba* leaves; and MA_ML, male *M. alba* leaves.

To characterize the composition and differences in microbial communities between male and female individuals across the four plant species, we analyzed endophytic bacterial communities at the phylum level and endophytic fungal communities at the genus level. Sex significantly influenced the composition of both endophytic bacterial and fungal communities (Fig. 2; Fig. S1 and S2 and Tables S3 and S4). Individuals of the same sex across plant species shared common bacterial and fungal taxa. A total of 19 bacterial phyla were shared among all female samples, including Abditibacteriota, Acidobacteria, and Actinobacteriota (Fig. 2A). The bacterial phylum Spirochaetota was detected in male *E. ulmoides* bark and in male *G. biloba* and *M. alba* leaves and was not detected in female samples across biological replicates. Across all female samples, 154 fungal genera were shared, including *Cladosporium* and *Uwebraunia* (Fig. 2B). The fungal genera *Albophoma* and *Striaticonidium*, as well as the species *Albophoma yamanashiensis*, *Striaticonidium brachysporum*, and *Hansfordia pruni*, were detected in female samples but were not detected in male samples across biological replicates. For example, *Albophoma yamanashiensis* was detected in female samples with a total occurrence of 15 out of 120 biological replicates (30 per species across four plant species), whereas it was absent in male samples. Seventeen bacterial phyla were shared across all male samples, including Actinobacteriota and Armatimonadota (Fig. 2C). s_uncultured_bacterium_g_Leucobacter and s_uncultured_bacterium_g_unclassified_c_AD3 were detected in male samples with no detection in females. Additionally, 142 fungal genera were shared across all male samples, including *Neodevriesia* and *Xenodevriesia* (Fig. 2D).

Proteobacteria and Actinobacteriota were the predominant bacterial phyla in the four dioecious plant species. Proteobacteria accounted for 24.59%–95.92% of the total bacterial sequences in these samples, while Actinobacteriota accounted for 2.95%–66.01%. The relative abundances of Proteobacteria were higher in female samples of *E. ulmoides* and *T. wallichiana* var. *mairei* barks and *M. alba* leaves compared with their male counterparts (Fig. 2E). *Sporidesmium* and an unclassified Dothideomycetes genus were the dominant fungal genera in *E. ulmoides* and *T. wallichiana* var. *mairei* bark. *Ramularia* and an unclassified Xylariales genus dominated in *G. biloba* leaves, whereas *Strelitziana* was the dominant fungal genus in *M. alba* leaves (Fig. 2F).

To investigate how sex and host species influence microbial community composition, we compared the relative abundances of common bacterial phyla and fungal genera across species and sexes. A total of 16 bacterial phyla were shared across *E. ulmoides* and *T. wallichiana* var. *mairei* barks and *G. biloba* and *M. alba* leaves (Fig. 2G). The top three most abundant bacterial phyla were Actinobacteriota (56.00%), Proteobacteria (39.15%), and Chloroflexi (2.46%). The relative abundances of these phyla varied among host species and sexes. For example, Actinobacteriota was the dominant phylum in *E. ulmoides* bark, accounting for 55.92% in female *E. ulmoides* bark and 66.01% in male *E. ulmoides* bark, but only 4.09% in female *G. biloba* leaves and 2.95% in male *G. biloba* leaves. In addition, 101 fungal genera were shared across *E. ulmoides* and *T. wallichiana* var. *mairei* barks and *G. biloba* and *M. alba* leaves, regardless of sex (Fig. 2H). *Sporidesmium* was the most abundant genus and exhibited variation across host species and sexes; for instance, it accounted for 27.68% in female *E. ulmoides* bark but only 13.84% in male *E. ulmoides* bark.

### Analysis of the beta diversity of endophytic bacterial and fungal communities

**Fig 3:**
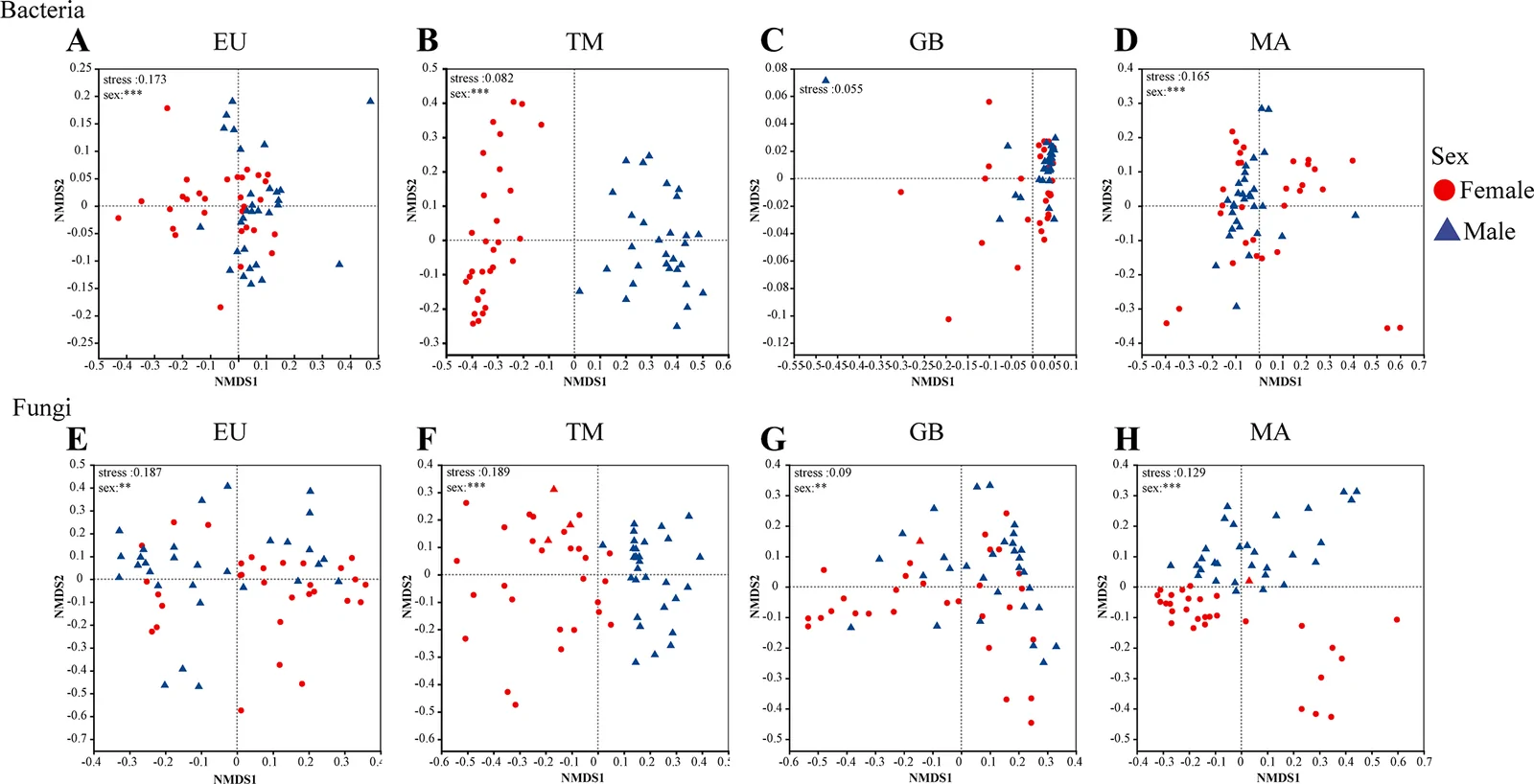
Effects of sex on bacterial (**A–D**) and fungal (**E–H**) community structures based on nonmetric multidimensional scaling analysis. PERMANOVA revealed significant effects of sex on both bacterial and fungal communities (*P < 0.05; **P < 0.01; and ***P < 0.001). EU, *E. ulmoides* bark; TM, *T. wallichiana* var. *mairei* bark; GB, *G. biloba* leaves; and MA, *M. alba* leaves.

**Fig 4:**
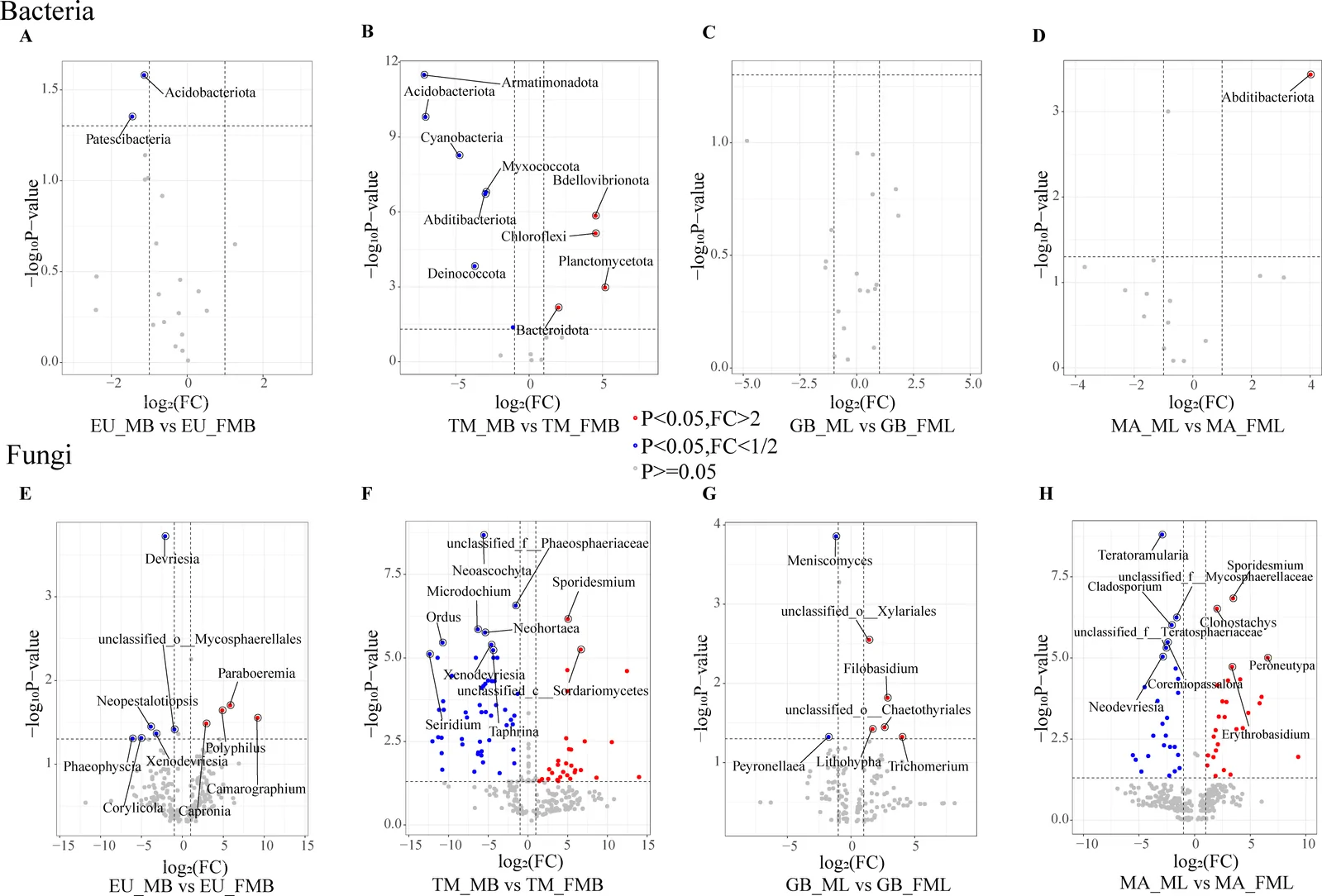
Volcano plots showing differential abundance of bacterial phyla (**A–D**) and fungal genera (**E–H**) between males and females for *E. ulmoides* (EU) and *T. wallichiana* var. *mairei* (TM) barks and *G. biloba* (GB) and *M. alba* (MA) leaves. Blue points indicate taxa significantly enriched in males; red points indicate taxa significantly enriched in females.

NMDS plots based on Bray–Curtis distances revealed distinct clustering of endophytic bacterial and fungal communities between male and female plants (Fig. 3). Notably, significant sex-associated clustering was observed in the bark of *E. ulmoides* and *T. wallichiana* var. *mairei* and in the leaves of *M. alba*, indicating that plant sex influences endophytic community structure. To identify the taxa contributing to these observed differences, we performed differential abundance analysis and summarized the results using volcano plots (Fig. 4). In *T. wallichiana* var. *mairei* bark, the abundances of bacteria such as Chloroflexi and Bacteroides were significantly higher in females than in males, whereas Armatimonadota, Acidobacteria, and Cyanobacteria were significantly elevated in males (Fig. 4B). The abundances of fungi such as *Paraboeremia* and *Polyphilus* were significantly higher in female *E. ulmoides* bark than in male *E. ulmoides* bark (Fig. 4E).

**Fig 5:**
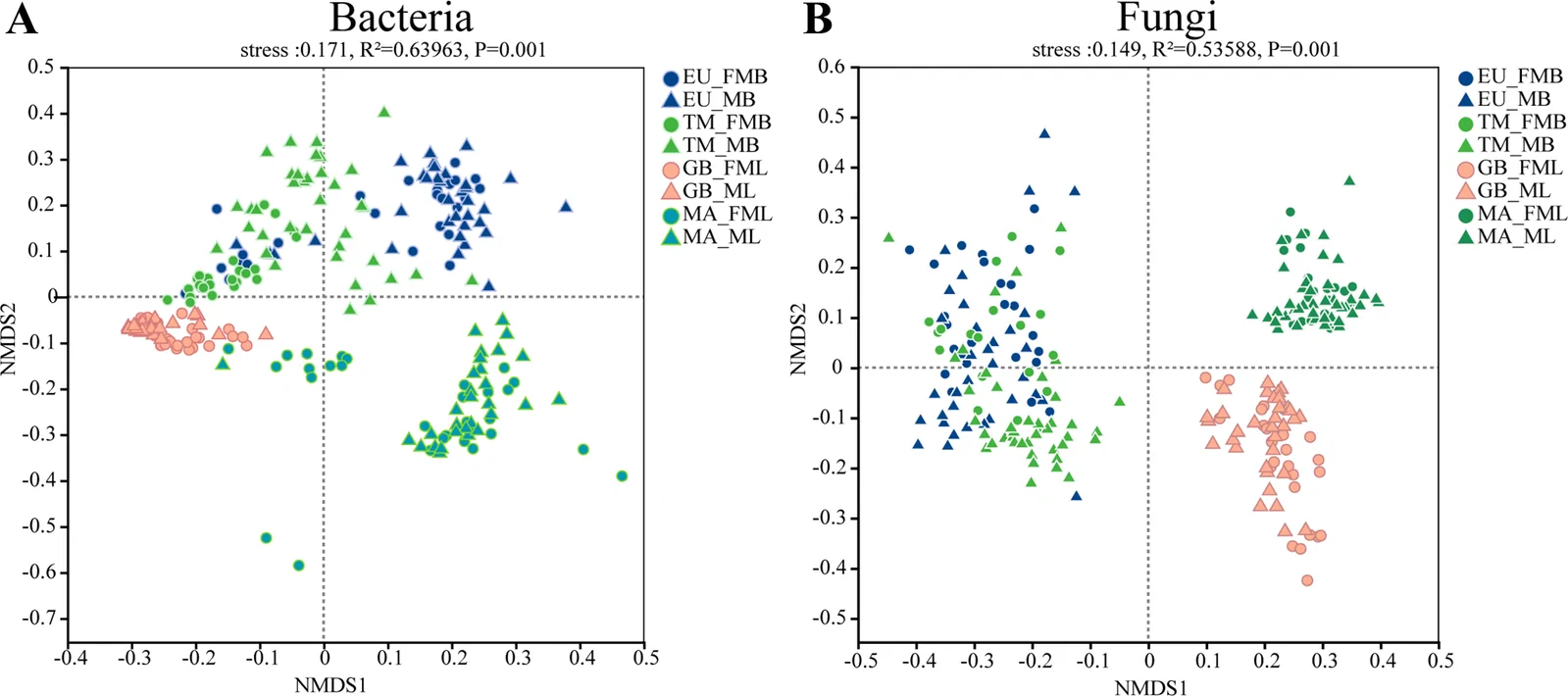
Effects of sex and host species on bacterial (**A**) and fungal (**B**) community structures based on NMDS analysis. PERMANOVA revealed significant effects of both sex and host species on bacterial and fungal communities. EU, *E. ulmoides* bark; TM, *T. wallichiana* var. *mairei* bark; GB, *G. biloba* leaves; and MA, *M. alba* leaves.

Although the types and number of differentially abundant bacteria and fungi between males and females vary across medicinal plant species, several consistent patterns emerge. For example, Acidobacteria was significantly enriched in male *E. ulmoides* and *T. wallichiana* var. *mairei* barks compared with females. *Sporidesmium* was one of the top three abundant taxa among differentially abundant endophytic fungi in *T. wallichiana* var. *mairei* barks and *M. alba* leaves, contributing significantly to sex-associated differences, with higher abundances in females than in males (Fig. 4). Endophytic bacterial and fungal communities were significantly influenced by host species, with host identity exerting a stronger effect than sex (Fig. 5). Furthermore, NMDS analysis revealed that these communities clustered according to host phylogeny, with gymnosperm hosts (*T. wallichiana* var. *mairei* bark and *G. biloba* leaves) distinctly separated from angiosperm hosts (*E. ulmoides* bark and *M. alba* leaves) (Fig. 5).

### Co-occurrence networks of endophytic bacteria and fungi

**Fig 6:**
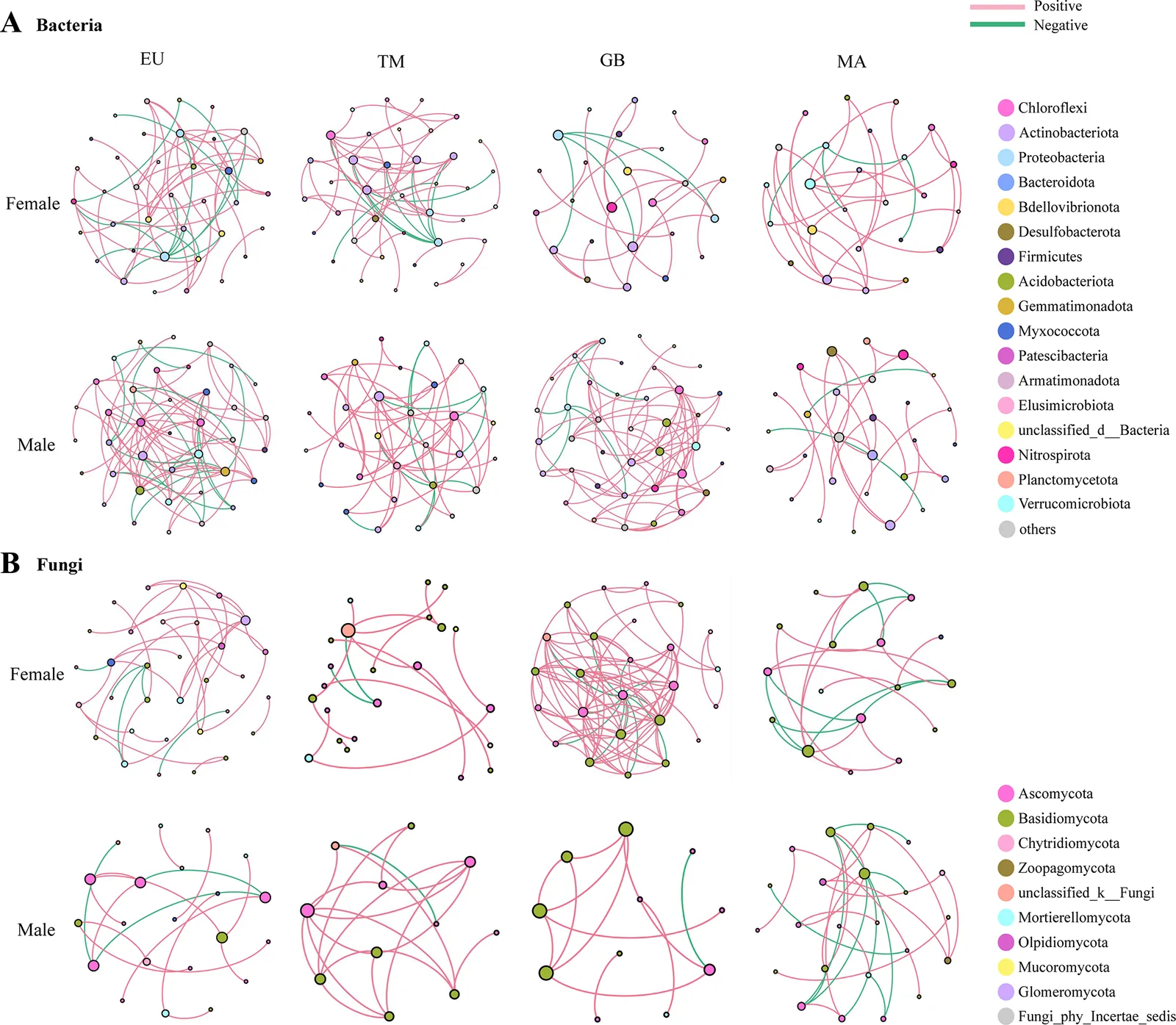
Differential effects of sex (female vs male) on the co-occurrence networks of endophytic bacterial (**A**) and fungal (**B**) communities within the *E. ulmoides* (EU) and *T. wallichiana* var. *mairei* (TM) barks and *G. biloba* (GB) and *M. alba* (MA) leaves. The colors of the dots in the network map indicate the phylum category, the dot size indicates the class level, the pink line indicates positive correlation, and the green line indicates negative correlation.

To further characterize the effects of host species and sex on plant endophytes, co-occurrence patterns of bacterial and fungal communities were assessed in male and female medicinal plants. Bacterial networks in male *E. ulmoides* and *T. wallichiana* var. *mairei* barks and *G. biloba* leaves exhibited higher average degrees than their female counterparts, with the highest average degree (4.256) observed in the bacterial network of male *G. biloba* leaves (Fig. 6A; Table S5). In contrast, fungal networks in female *E. ulmoides* bark and female *G. biloba* and *M. alba* leaves displayed higher average degrees compared with those in males (Fig. 6B; Table S5). Notably, female *G. biloba* leaves exhibited the highest network complexity, characterized by the highest average degree (6.8) and clustering coefficient (0.71) (Fig. 6B; Table S5). Across all samples, co-occurrence links between Actinobacteriota and other phyla—and between Proteobacteria and other phyla—are stronger than those among the remaining phyla (Fig. 6).

### Predictive function of endophytic bacteria and fungi

**Fig 7:**
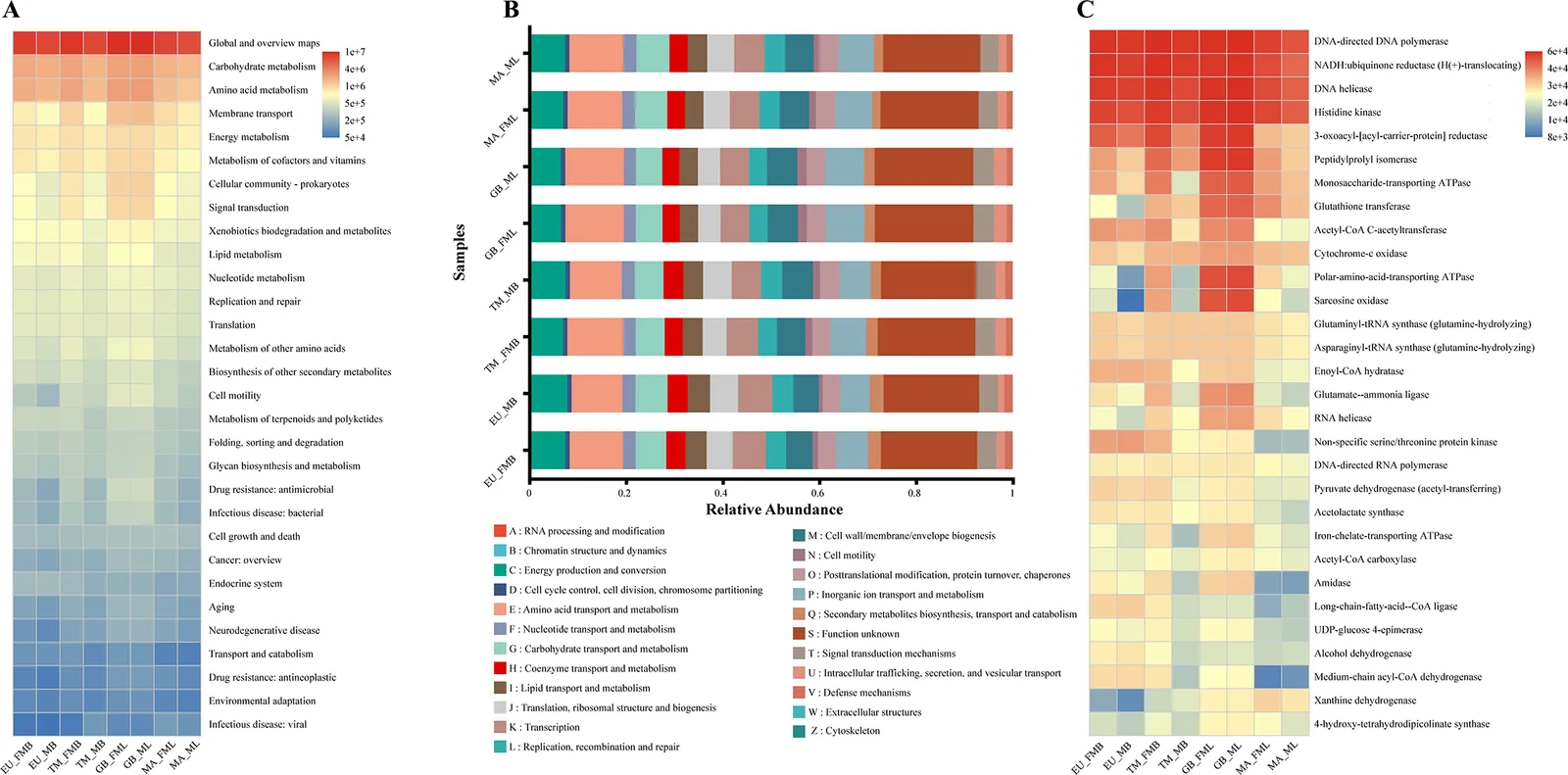
Functional prediction and classification of bacterial communities. (**A**) Heatmap of bacterial gene functions predicted by PICRUSt2, with rows representing KEGG pathways and columns representing samples. (**B**) Relative abundances of COG functional categories in dominant endophytic bacteria from *E. ulmoides* and *T. wallichiana* var. *mairei* barks and *G. biloba* and *M. alba* leaves. (**C**) Heatmap of fungal enzyme function predicted by PICRUSt2, with rows representing enzymes and columns representing samples. EU_FMB, female *E. ulmoides* bark; EU_MB, male *E. ulmoides* bark; TM_FMB, female *T. wallichiana* var. *mairei* bark; TM_MB, male *T. wallichiana* var. *mairei* bark; GB_FML, female *G. biloba* leaves; GB_ML, male *G. biloba* leaves; MA_FML, female *M. alba* leaves; and MA_ML, male *M. alba* leaves.

Endophytic bacterial communities from the four dioecious plant species exhibited broadly similar predicted functional profiles across major metabolic pathways. Global and overview maps, carbohydrate metabolism, and amino acid metabolism were among the most abundant predicted categories in both male and female plants. However, sex-associated differences were observed in *E. ulmoides*, *T. wallichiana* var. *mairei*, and *M. alba*, where females exhibited relatively higher predicted abundances of functions related to energy metabolism, metabolism of cofactors and vitamins, glycan biosynthesis and metabolism, and aging (Fig. 7A). Overall, endophytic bacterial communities exhibited broad functional potential, with genes involved in metabolism, environmental adaptation, genetic information processing, cellular functions, and uncharacterized proteins functions widely represented. Functions related to energy production/conversion and amino acid transport/metabolism were among the most frequently predicted categories (Fig. 7B). It should be emphasized that these results represent predicted functional potential rather than experimentally validated activities. At the enzyme level, predicted functions of bacterial communities were broadly conserved across samples, including DNA-directed DNA polymerase, NADH: ubiquinone (H^+^)-translocating reductase, DNA helicase, and histidine kinase. Nevertheless, some sex-associated differences were observed in *E. ulmoides* and *T. wallichiana* var. *mairei* barks and *M. alba* leaves, where females exhibited relatively higher predicted abundances of enzymes such as 3-oxoacyl-[acyl-carrier-protein] reductase, peptidylprolyl isomerase, monosaccharide-transporting ATPase, glutathione transferase, acetyl-CoA C-acetyltransferase, polar-amino-acid-transporting ATPase, sarcosine oxidase, and others (Fig. 7C). Endophytic fungal functional profiles were also predicted to be relatively similar across samples, with enzymes such as ATPases, glucan 1,4-alpha-glucosidase, and DNA-directed RNA polymerase showing higher predicted abundances in both male and female hosts (Fig. S4).

## DISCUSSION

This study revealed significant sex effects on bacterial diversity, with a higher Shannon index observed in males of *E. ulmoides*, *T. wallichiana* var. *mairei*, and *M. alba* compared with females (Fig. 1A through D). Sexual dimorphism in morphology, carbon allocation, secondary metabolite production, and nutrient uptake can create distinct internal niches for microbial colonization (7, 17, 42). For example, male and female *P. cathayana* exhibited differences in internal starch content, pH, and metabolic profiles that correlated with bacterial richness (16). Male plants often display greater physiological stability and stress tolerance, which may support a more diverse and resilient microbiota by providing a more consistent internal environment for microbial recruitment and persistence (43). Moreover, sex-related differences in carbon allocation, metabolite production, and nutrient availability can generate distinct internal environments that differentially favor bacterial colonization, potentially leading to broader ecological niches and increased diversity in males. However, the Shannon index of fungal diversity was not significantly affected by sex (Fig. 1E through H). Sex-related differences were greater in bacterial than fungal communities, likely due to multiple ecological and physiological mechanisms. First, endophytic bacterial assembly is often more strongly shaped by host factors and physiological status, while fungal communities are more influenced by microclimate and dispersal of exogenous spores (44, 45). Second, bacteria generally have shorter generation times and greater sensitivity to specific carbon sources and metabolites, so they may respond more rapidly and strongly than fungi to sex-driven changes in biochemical microenvironments (46). By contrast, fungal endophytes may be more affected by developmental stage or microhabitat structure than by plant sex, producing more homogeneous diversity patterns (47).

Plant sex had no effect on the distribution of the predominant bacterial and fungal phyla in *E. ulmoides*, *T. wallichiana* var. *mairei*, *G. biloba*, and *M. alba*, supporting the presence of conserved core endophytic bacterial and fungal communities shared among the different plant hosts (48–50). In this study, the most prevalent bacterial phyla (Actinobacteriota and Proteobacteria) and fungal phyla (Ascomycota and Basidiomycota) were shared across all samples (Fig. 2). Across samples, Actinobacteriota and Proteobacteria co-occur more strongly with other phyla than others do (Fig. 6). Actinobacteriota and Proteobacteria are dominant members of endophytic bacterial communities across plant species (48, 51, 52). Actinobacteriota typically account for 10%–40% of endophytic bacterial communities, whereas Proteobacteria constitute 30%–70% (48, 52). These phyla exhibit strong adaptability and multifunctionality, including organic matter degradation, plant growth promotion, and pathogen suppression, enabling them to maintain stability in the complex and dynamic plant endophytic environment and provide ecological services to host plants (53, 54). Meanwhile, Ascomycota and Basidiomycota constitute the core fungal phyla in plant endophytic communities, with Ascomycota typically accounting for 60%–90% and Basidiomycota for 5%–30% of endophytic fungal communities (48). Ascomycota and Basidiomycota are known to confer tolerance to biotic and abiotic stresses, enhance nutrient uptake, and produce bioactive metabolites (55, 56). These functional advantages likely promote their successful colonization and persistence within plant tissues, allowing them to dominate endophytic fungal communities across host species and sexes. However, although plant sex did not significantly influence the predominant bacterial and fungal phyla, sex-specific differences were still observed in certain bacterial phyla and fungal taxa. In this study, Spirochaetota was detected in male samples of *E. ulmoides*, *G. biloba*, and *M. alba* but not in female samples across biological replicates. This may be attributed to the higher primary metabolic activity and nutrient demands usually exhibited by male plants (57). Male plants allocate more resources to growth rather than reproduction, which may create nutrient-rich environments favorable for microbial colonization (58). Furthermore, Spirochaetota are anaerobic or microaerophilic and thrive in environments with lower oxygen availability, which could be more prevalent in male plants due to their higher metabolic rates (59). Our study provides the first report of *Albophoma* and *Striaticonidium* being detected in female *E. ulmoides*, *T. wallichiana* var. *mairei*, *G. biloba*, and *M. alba* but not in male samples. This may be due to sex-specific differences in plant physiology, secondary metabolite production, and resource allocation.

Sex acts as a biological filter during microbial colonization, resulting in sex-specific enrichment of certain endophytic bacteria and fungi in male and female plants (16, 60). *P. cathayana* trees exhibit distinct microbial communities in male and female individuals (16). In this study, Acidobacteria was significantly enriched in males compared with females in *E. ulmoides* and *T. wallichiana* var. *mairei* (Fig. 4). Members of Acidobacteria are known for their ability to degrade complex organic matter, regulate environmental pH, and enhance resistance to heavy metals, thereby contributing to microbial survival under stress conditions (61). Male plants often exhibit greater tolerance to abiotic stresses, such as drought and salinity, than female plants (7, 62). Therefore, the enrichment of Acidobacteria in male plants may reflect a sex-associated microbial strategy that enhances host adaptability to adverse environments. Moreover, volcano plots consistently identified *Sporidesmium* as a female-associated fungal taxon in *E. ulmoides* and *T. wallichiana* var. *mairei*, with significantly higher levels in females than in males. In dioecious plants, female individuals generally incur higher reproductive costs due to fruit and seed development, requiring sustained investment in carbon resources, nutrient acquisition, and tissue protection throughout the reproductive cycle (63, 64). *Sporidesmium* is a saprobic or lignicolous fungus capable of degrading complex organic substrates and producing bioactive secondary metabolites, which may contribute to local nutrient turnover and microbial-mediated defense (65). Such sex-specific enrichment suggests that differences in reproductive investment and tissue microenvironments between males and females act as biological filters shaping endophytic fungal community assembly at finer taxonomic levels (16, 48, 60). Altogether, these findings highlight that plant sex not only influences the relative abundances of specific bacterial and fungal taxa but also drives the differential recruitment of functionally distinct microbial lineages. Host tissue type is an important determinant of endophytic microbial composition. In this study, bark was sampled from *E. ulmoides* and *T. wallichiana* var. *mairei*, while leaves were sampled from *G. biloba* and *M. alba*. This design may introduce variation unrelated to plant sex; therefore, although our results indicate sex-associated differences within individual species, cross-species comparisons remain limited. Future studies using standardized tissue sampling across species will be necessary to determine whether conserved sex-associated microbial patterns exist in dioecious plants.

Endophytic microorganisms and their host plants are considered to have undergone long-term co-evolution (48, 66). In our study, endophytic bacterial and fungal communities tended to cluster according to host phylogeny, with gymnosperm hosts (*T. wallichiana* var. *mairei* bark and *G. biloba* leaves) separated from angiosperm hosts (*E. ulmoides* bark and *M. alba* leaves) (Fig. 5). This finding is consistent with recent research on plant microbiome assembly, suggesting that plant microbiomes are largely influenced by the host plant’s phylogeny. Plant microbiomes are shaped not only by vertical transmission but also by the joint influence of host genotype and environmental context (67). Specifically, during the evolutionary process, the phylogenetic relationship between plants is often closely linked to their microbiome composition, with species in the same family exhibiting more similar microbial communities, while distantly related plants harbor more distinct microbial structures. This phenomenon is known as phylosymbiosis (68, 69). For example, plants with closer phylogenetic relationships tend to harbor more similar microbiomes, particularly under controlled environments (70). This phylogenetic association is also evident in our study, where gymnosperms and angiosperms exhibited distinct microbial community patterns. As two deeply diverged evolutionary lineages in the plant kingdom, gymnosperms and angiosperms were significantly different in tissue structure, metabolic characteristics, and defense mechanisms (71, 72). These differences likely act as long-term ecological filters that shape their respective microbial community structures. In addition, horizontal gene transfer from microbiomes to host plants may play a role in influencing the genomic landscape of host plants. For example, the frequent identification of microbial terpene synthase-like genes in non-seed land plants provides further evidence of horizontal gene transfer from the microbiome, which may contribute to altering the genomic composition and functional capabilities of the host plant (73). Therefore, our findings support a possible role for plant evolution in shaping endophytic microbial communities and suggest that plant evolution may contribute to differences in microbiome distribution across host lineages.

### Conclusions

This study investigated the assembly of endophytic bacterial and fungal communities in male and female individuals of four dioecious plant species (*E. ulmoides*, *T. wallichiana* var. *mairei*, *G. biloba*, and *M. alba*). The results demonstrate that plant sex significantly influences microbial community composition, as evidenced by significantly higher bacterial alpha diversity (Shannon index) in *E. ulmoides*, *T. wallichiana* var. *mairei*, and *M. alba* males compared with females, as well as by sex-specific differences in specific bacterial and fungal taxa. Spirochaetota was detected in male samples of *E. ulmoides*, *G. biloba*, and *M. alba* but not in female samples across biological replicates. In contrast, the fungal genera *Albophoma* and *Striaticonidium* were detected only in female individuals across all four species. Despite these sex-associated differences, the predominant taxonomic composition of endophytic bacteria and fungi remained largely conserved between sexes across all hosts. Proteobacteria, Actinobacteriota, Ascomycota, and Basidiomycota were consistently shared across *E. ulmoides*, *T. wallichiana* var. *mairei*, *G. biloba*, and *M. alba*. Co-occurrence network analysis further revealed that these dominant phyla exhibited stronger associations with other OTUs, supporting the presence of a conserved core endophytic microbiome shared across host species and sexes. These results enhance understanding of sex-associated endophytic communities in dioecious plants and may inform studies on plant sex differentiation and potential plant–microbiome associations.

## Data Availability

The raw sequencing reads were deposited in the NCBI Sequence Read Archive (SRA) database under the following accession number: PRJNA1464396.

## SUPPLEMENTAL MATERIAL

Supplemental figures spectrum.00976-26-s0001.docx Fig. S1 to S4.

Supplemental legends spectrum.00976-26-s0002.docx Legends for Tables S1 to S4.

Table S5 spectrum.00976-26-s0003.docx Differential effects of sex (female vs. male) on endophytic bacterial and fungal co-occurrence in *E. ulmoides* (EU) and *T. wallichiana var. mairei* (TM) barks and *G. biloba* (GB) and *M. alba* (MA) leaves.

Tables S1 spectrum.00976-26-s0004.xlsx OTU abundance matrix from 16S rRNA gene sequencing of endophytic bacterial communities.

Tables S2 spectrum.00976-26-s0005.xlsx OTU abundance matrix from ITS sequencing of endophytic fungal communities.

Tables S3 spectrum.00976-26-s0006.xlsx Bacterial phyla used in Venn diagram analysis.

Tables S4 spectrum.00976-26-s0007.xlsx Fungal genera used in Venn diagram analysis.

ASM does not own the copyrights to Supplemental Material that may be linked to, or accessed through, an article. The authors have granted ASM a non-exclusive, world-wide license to publish the Supplemental Material files. Please contact the corresponding author directly for reuse.
