# Supplementary material for: Impact of plant sex on endophytic microbial diversity and community assembly in dioecious species
Source: Microbiol Spectr. 2026 Sep 22;14(11):e00976-26. doi: 10.1128/spectrum.00976-26 (PMC13729149; doi:10.1128/spectrum.00976-26)
Supplement: Supplementary file 1 [file spectrum.00976-26-s0001.docx]

Figure S1. Rarefaction curves for bacterial (a) and fungal (b) operational taxonomic units. EU_FMB, female *E. ulmoides* bark; EU_MB, male *E. ulmoides* bark; TM_FMB, female *T. wallichiana* var. *mairei* bark; TM_MB, male *T. wallichiana* var. *mairei* bark; GB_FML, female *G.* *biloba* leaves; GB_ML, male *G.* *biloba* leaves; MA_FML, female *M. alba* leaves; MA_ML, male *M. alba* leaves.

Figure S2. Upset Plots showing the shared and unique bacterial phyla (a–c) and fungal genera (d–f) among male and female samples of *E. ulmoides*, *T. wallichiana* var. *mairei*, *G. biloba*, and *M. alba*. EU_FMB, female *E. ulmoides* bark; EU_MB, male *E. ulmoides* bark; TM_FMB, female *T. wallichiana* var. *mairei* bark; TM_MB, male *T. wallichiana* var. *mairei* bark; GB_FML, female *G.* *biloba* leaves; GB_ML, male *G.* *biloba* leaves; MA_FML, female *M. alba* leaves; MA_ML, male *M. alba* leaves.

Figure S3. Venn diagrams showing the shared and unique bacterial phyla (a–d) and fungal genera (e–h) among male and female samples of *E. ulmoides*, *T. wallichiana* var. *mairei*, *G. biloba*, and *M. alba*. EU_FMB, female *E. ulmoides* bark; EU_MB, male *E. ulmoides* bark; TM_FMB, female *T. wallichiana* var. *mairei* bark; TM_MB, male *T. wallichiana* var. *mairei* bark; GB_FML, female *G.* *biloba* leaves; GB_ML, male *G.* *biloba* leaves; MA_FML, female *M. alba* leaves; MA_ML, male *M. alba* leaves.

Figure S4. Functional prediction and classification of fungal communities. Heatmap of fungal enzyme functions predicted by PICRUSt2, with rows representing enzymes and columns representing samples. EU_FMB, female *E. ulmoides* bark; EU_MB, male *E. ulmoides* bark; TM_FMB, female *T. wallichiana* var. *mairei* bark; TM_MB, male *T. wallichiana* var. *mairei* bark; GB_FML, female *G.* *biloba* leaves; GB_ML, male *G.* *biloba* leaves; MA_FML, female *M. alba* leaves; MA_ML, male *M. alba* leaves.
