# Supplementary material for: Impact of plant sex on endophytic microbial diversity and community assembly in dioecious species
Source: Microbiol Spectr. 2026 Sep 22;14(11):e00976-26. doi: 10.1128/spectrum.00976-26 (PMC13729149; doi:10.1128/spectrum.00976-26)
Supplement: Supplementary file 2 [file spectrum.00976-26-s0002.docx]

Legends for Supplemental Tables 1-4

Table S1. OTU abundance matrix from 16S rRNA gene sequencing of endophytic bacterial communities.

This table lists bacterial operational taxonomic units (OTUs) as rows and individual samples as columns. Each cell shows the number of sequences (abundance) of a given OTU detected in that sample. FMb_ZY: EU_FMB, female *E. ulmoides* bark; Mb_ZY: EU_MB, male *E. ulmoides* bark; Fb_LS: TM_FMB, female *T. wallichiana* var. *mairei* bark; Mb_LS: TM_MB, male *T. wallichiana* var. *mairei* bark; FML_PZ: GB_FML, female *G.* *biloba* leaves; ML_PZ: GB_ML, male *G.* *biloba* leaves; FML_XS: MA_FML, female *M. alba* leaves; ML_XS8: MA_ML, male *M. alba* leaves.

Table S2. OTU abundance matrix from ITS sequencing of endophytic fungal communities.

This table lists fungal operational taxonomic units (OTUs) as rows and individual samples as columns. Each cell shows the number of sequencing reads assigned to that OTU in the corresponding sample. FMb_ZY: EU_FMB, female *E. ulmoides* bark; Mb_ZY: EU_MB, male *E. ulmoides* bark; Fb_LS: TM_FMB, female *T. wallichiana* var. *mairei* bark; Mb_LS: TM_MB, male *T. wallichiana* var. *mairei* bark; FML_PZ: GB_FML, female *G.* *biloba* leaves; ML_PZ: GB_ML, male *G.* *biloba* leaves; FML_XS: MA_FML, female *M. alba* leaves; ML_XS8: MA_ML, male *M. alba* leaves.

Table S3. Bacterial phyla used in Venn diagram analysis.

This table lists the bacterial phyla detected in each sample group that were included in the Venn diagram analysis. EU_FMB, female *E. ulmoides* bark; EU_MB, male *E. ulmoides* bark; TM_FMB, female *T. wallichiana* var. *mairei* bark; TM_MB, male *T. wallichiana* var. *mairei* bark; GB_FML, female *G.* *biloba* leaves; GB_ML, male *G.* *biloba* leaves; MA_FML, female *M. alba* leaves; MA_ML, male *M. alba* leaves.

Table S4. Fungal genera used in Venn diagram analysis.

This table lists the fungal genera detected in each sample group that were included in the Venn diagram analysis. EU_FMB, female *E. ulmoides* bark; EU_MB, male *E. ulmoides* bark; TM_FMB, female *T. wallichiana* var. *mairei* bark; TM_MB, male *T. wallichiana* var. *mairei* bark; GB_FML, female *G.* *biloba* leaves; GB_ML, male *G.* *biloba* leaves; MA_FML, female *M. alba* leaves; MA_ML, male *M. alba* leaves.
