# Supplementary material for: Impact of plant sex on endophytic microbial diversity and community assembly in dioecious species
Source: Microbiol Spectr. 2026 Sep 22;14(11):e00976-26. doi: 10.1128/spectrum.00976-26 (PMC13729149; doi:10.1128/spectrum.00976-26)
Supplement: Supplementary file 3 [file spectrum.00976-26-s0003.docx]

Table S5. Differential effects of sex (female vs. male) on endophytic bacterial and fungal co-occurrence in *E. ulmoides* (EU) and *T. wallichiana* var. *mairei* (TM) barks and *G. biloba* (GB) and *M. alba* (MA) leaves.

|  | **Positive edge** | **Negative edge** | **Average degree** | **Modularity** | | **Average clustering coefficient** | **Average path distance** |
| --- | --- | --- | --- | --- | --- | --- | --- |
| **Bacteria** |  |  |  |  |  | |  |
| EU |  |  |  |  |  | |  |
| Female | 40 | 15 | 2.973 | 0.514 | 0.335 | | 3.233 |
| Male | 61 | 19 | 4.211 | 0.526 | 0.549 | | 2.822 |
| TM |  |  |  |  |  | |  |
| Female | 46 | 9 | 2.895 | 0.573 | 0.481 | | 2.356 |
| Male | 41 | 10 | 3.4 | 0.606 | 0.644 | | 2.422 |
| GB |  |  |  |  |  | |  |
| Female | 22 | 4 | 2.08 | 0.683 | 0.365 | | 3.632 |
| Male | 76 | 7 | 4.256 | 0.534 | 0.582 | | 3.382 |
| MA |  |  |  |  |  | |  |
| Female | 27 | 4 | 2.48 | 0.688 | 0.575 | | 2.974 |
| Male | 22 | 2 | 1.714 | 0.727 | 0.133 | | 4.291 |
| **Fungi** |  |  |  |  |  | |  |
| EU |  |  |  |  |  | |  |
| Female | 32 | 5 | 2.312 | 0.584 | 0.295 | | 4.534 |
| Male | 14 | 3 | 1.619 | 0.715 | 0.125 | | 2.411 |
| TM |  |  |  |  |  | |  |
| Female | 14 | 2 | 1.391 | 0.744 | 0 | | 2 |
| Male | 19 | 1 | 3.636 | 0.211 | 0.492 | | 1.927 |
| GB |  |  |  |  |  | |  |
| Female | 73 | 12 | 6.8 | 0.224 | 0.71 | | 1.675 |
| Male | 11 | 1 | 2.182 | 0.302 | 0.633 | | 1.696 |
| MA |  |  |  |  |  | |  |
| Female | 15 | 9 | 2.824 | 0.44 | 0.465 | | 2.309 |
| Male | 16 | 9 | 2.174 | 0.597 | 0.403 | | 3.104 |
